# RECRUITMENT TO THE GERONTOLOGY CERTIFICATE: STRATEGIES FROM UTAH STATE UNIVERSITY

**DOI:** 10.1093/geroni/igz038.2285

**Published:** 2019-11-08

**Authors:** Elizabeth B Fauth, Yin Liu

**Affiliations:** Utah State University, Logan, Utah, United States

## Abstract

There is a workforce shortage in age-related fields, in both medical and non-medical, and professional and nonprofessional employment levels. Jobs in aging-related fields are cited as growth careers, or in lists of “top ten growing jobs”, yet academic training programs in gerontology are still facing low enrollment and other long-standing barriers. Utah State University offers an interdisciplinary gerontology certificate housed within the department of Human Development and Family Studies (HDFS). Recruitment to the certificate is most effective from two “feeder” classes: Adult Development and Aging (in HDFS), and via a guest lecture in Community Health (in Kinesiology and Health Sciences). Face-to-face meetings with academic advisors in other departments (e.g. Nutrition) has resulted in referrals to the certificate program. Office hours and drop-ins from interested students falls on the certificate coordinator, but is essential in recruitment. In sum, face-to-face recruitment is time intensive, but significantly more effective than online/print material.

